# ParSCo: celebrating 10 years of a unique parasitology summer course

**DOI:** 10.1186/s13071-024-06174-z

**Published:** 2024-02-26

**Authors:** Filipe Dantas-Torres, Marcos Antonio Bezerra-Santos, Jairo Alfonso Mendoza-Roldan, Riccardo Paolo Lia, Livia Perles, Juan Pedro Barrera, Renata Fagundes-Moreira, Mariaelisa Carbonara, Antonio Varcasia, Emanuele Brianti, Georgiana Deak, Alicia Rojas, Guadalupe Miró, Petr Volf, Gad Baneth, Domenico Otranto

**Affiliations:** 1 Aggeu Magalhães Institute, Fundação Oswaldo Cruz (Fiocruz), Recife, Brazil; 2 Department of Veterinary Medicine, University of Bari “Aldo Moro”, Valenzano, Italy; 3 Animal Health Department, Veterinary Faculty, Universidad Complutense de Madrid, Madrid, Spain; 4 Department of Veterinary Medicine, University of Sassari, Sassari, Italy; 5 Department of Veterinary Sciences, University of Messina, Messina, Italy; 6 Department of Parasitology and Parasitic Diseases, Faculty of Veterinary Medicine, University of Agricultural Sciences and Veterinary Medicine of Cluj-Napoca, Cluj-Napoca, Romania; 7 Laboratory of Helminthology, Faculty of Microbiology, University of Costa Rica, San Jose, Costa Rica; 8 Department of Parasitology, Faculty of Science, Charles University, Prague, Czech Republic; 9 Koret School of Veterinary Medicine, Hebrew University of Jerusalem, Rehovot, Israel; 10 Department of Veterinary Clinical Sciences, City University of Hong Kong, Kowloon, Hong Kong

## Abstract

ParSCo (Parasitology Summer Course) is an intense, 1-week-long summer course organized by the Parasitology Unit of the Department of Veterinary Medicine, University of Bari, Italy, with the support of the World Association for the Advancement of Veterinary Parasitology (WAAVP), the European Veterinary Parasitology College (EVPC) and *Parasites and Vectors*. The course, which is conducted in southern Italy, is planned for parasitologists and post-graduate students working in the field of parasitology. The course consists of theoretical and practical lessons, which include the collection, identification and diagnosis of parasites of pets, livestock and wildlife. The participants in ParSCo are afforded the opportunity to be involved in clinical examination and sample collection for the diagnosis of parasitic diseases (e.g. leishmaniosis, thelaziosis and many tick-borne diseases) present in the Mediterranean Basin. The course is conducted at *Casa di Caccia*, a hunting lodge situated in the Gallipoli Cognato Forest near the Basento River in the Basilicata region in southern Italy. In addition to the training purpose, ParSCo is a great opportunity for sharing knowledge and expertise while becoming part of the parasitology community in a pleasant environment. In this editorial, we share some information and celebrate 10 years of ParSCo, looking forward to forthcoming sessions of this unique parasitology summer course.

**Graphical Abstract:**

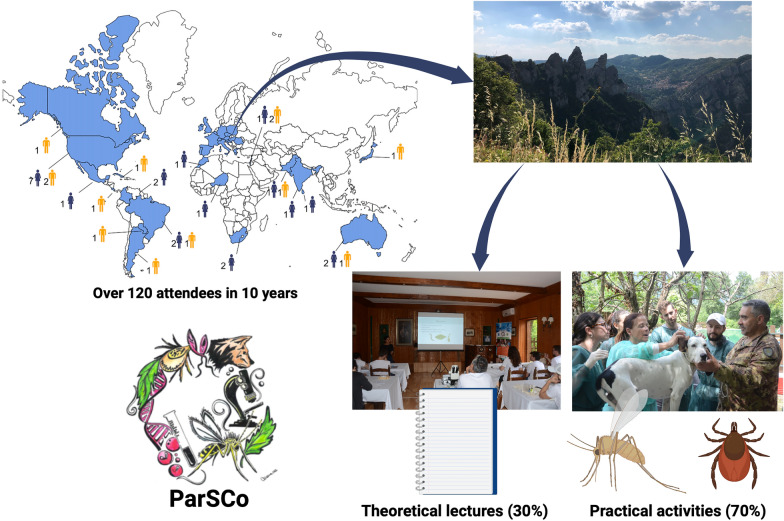

## ParSCo: a brief history

ParSCo, “**Par**asitology **S**ummer **Co**urse,” is an annual summer course organized by the Parasitology Unit of the Department of Veterinary Medicine, University of Bari, Italy. It is an intense, 1-week-long course for parasitologists and post-graduate students working in the field of veterinary parasitology. For more details, see a promotional video at the following LINK. https://www.youtube.com/watch?v=qpZ6FV9KQVI.

The course is mostly focused on practical activities, with theoretical lectures making up around 30% of the whole program. Practical activities include the collection, identification and diagnosis of parasites in pets, livestock and wildlife. Particular emphasis is placed on the collection and identification of ticks and sand flies. Attendees also can participate in clinical examination and sample collection for the diagnosis of parasitic diseases, including leishmaniosis and eyeworm disease.

The course traditionally takes place in Basilicata in southern Italy, in the heart of the Mediterranean region. This area has received significant attention from researchers not only for its outstanding species richness but also because it represents an ideal model for the study of parasites and parasitic diseases. Indeed, this region has been a suitable site for many research projects [1–23]. The only exception was ParSCo VIII, co-organized with the Parasitology Unit of the Department of Veterinary Sciences of the University of Messina, Italy, held in the Aeolian Archipelago, a magnificent landscape located in the Tyrrhenian Sea off the northern coast of Sicily, Italy, composed of seven islands of volcanic origin. The islands are home to unique animal species, and a considerable number of parasites, inhabiting different microenvironments, can be found in Lipari, Vulcano, Salina and Stromboli. In this article, we present information on this unique course, which is celebrating 10 years of history.

## Location and infrastructure: the park and hunting lodge

**Fig. 1:**
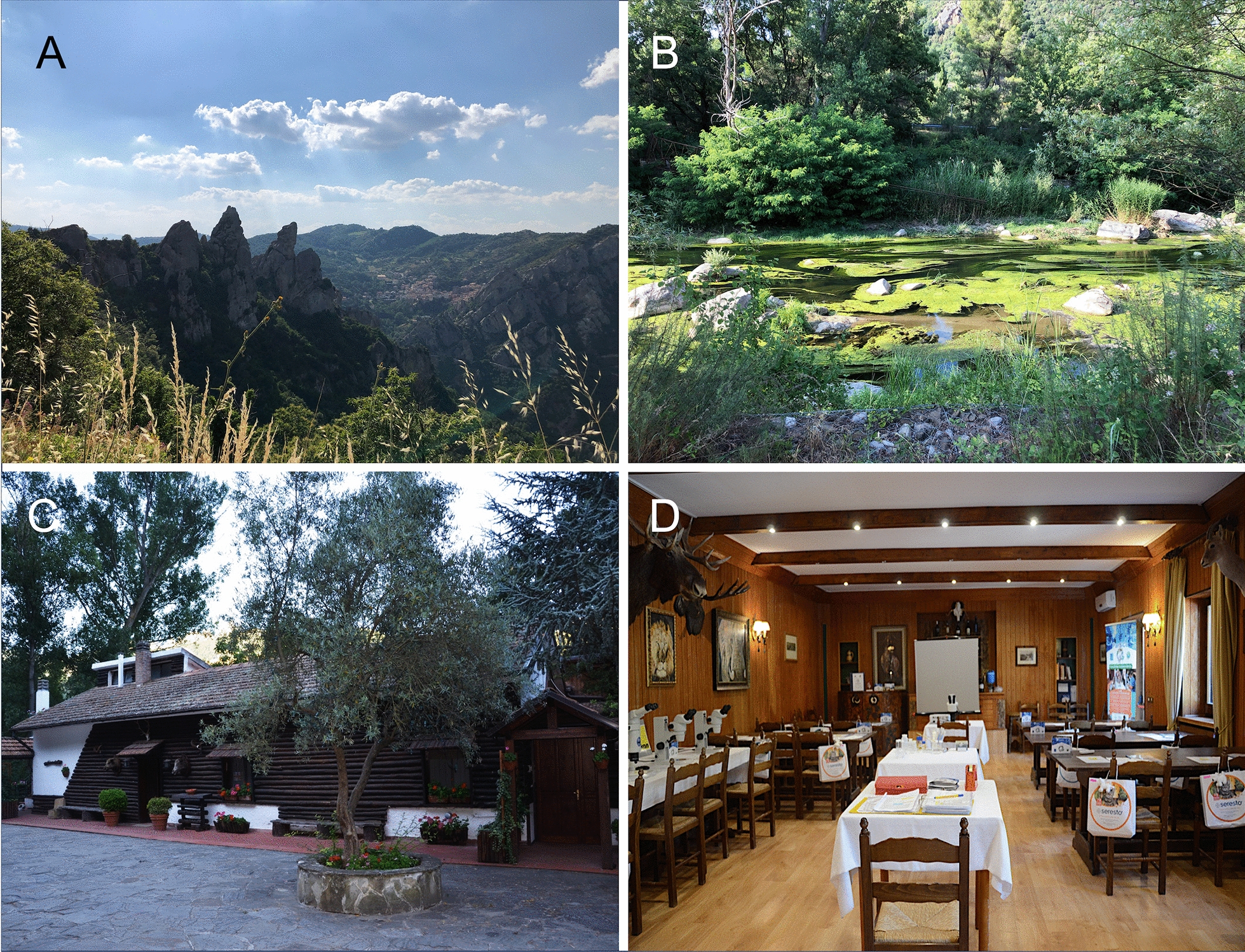
Parco Regionale di Gallipoli Cognato e delle Piccole Dolomiti Lucane. **a** Ridges of arenaceous rock (the “Dolomiti Lucane”); **b** a stream of the Basentum river; **c** the venue of the summer course (La Casa di Caccia); **d** the main teaching room, used for both oral and practical lessons

The “Parco Regionale di Gallipoli Cognato e delle Piccole Dolomiti Lucane” (40°31ʹ57″N, 16°07ʹ04″E) covers an area of 27.027 hectares within the borders of the towns of Accettura, Calciano and Oliveto Lucano in the province of Matera and Castelmezzano and Pietrapertosa in the province of Potenza. The park protects and safeguards a large area at the center of Basilicata’s regional territory. Among the most significant natural elements are two ridges of arenaceous rock (Fig. 1a). The presence of watercourses is conspicuous (Fig. 1b) in the form of seasonal torrents and springs. The wild fauna include wild boars, wolves, foxes, badgers, porcupines, wildcats and many other animal species.

The *Casa di Caccia* (https://lacasadicaccia.it/) is a hunting lodge located in the Gallipoli Cognato Forest near the Basento River banks. Its rustic architecture is reminiscent of a wooden chalet built among the green mountains (Fig. 1c). The lodge has several apartments with a total of 25 beds, a restaurant and a swimming pool. In addition, two classrooms have been set up inside the lodge, one for lectures and the other for practical lessons, where microscopes and stereomicroscopes (Fig. 1d) are available for ParSCo attendees.

## Lectures and practical activities: from theory to practice

The field of parasitology is a discipline that requires high expertise, critical thinking and constant updating. For these reasons, a combination of practical and theoretical activities is essential for the acquisition of the necessary skills in parasitology [24]. The ParSCo program combines oral talks by experts in specific subjects such as vector-borne pathogens; tick, fly and sand fly ecology and taxonomy; clinical features and management of vector-borne diseases; and helminth taxonomy and identification. After each theoretical lecture, a practical activity in the field, with demonstrations of sick animals and mounted parasitic specimens, is performed to integrate concepts and put them into practice.

**Fig. 2:**
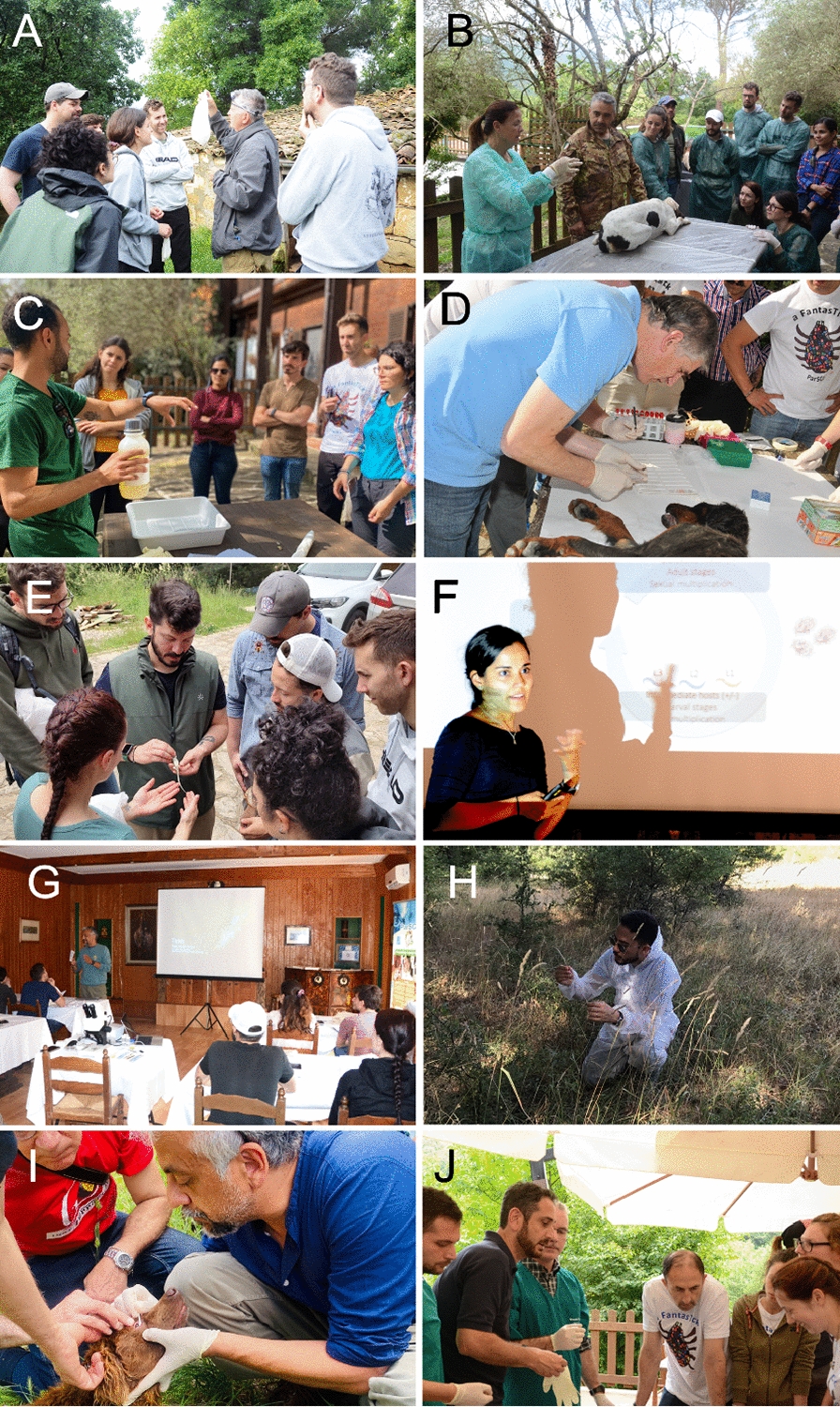
Lecturers at ParSCo. **a** Petr Volf showing the sand flies captured with a CDC light trap; **b** Guadalupe Miró conducting a physical examination of a dog; **c** Marcos Antonio Santos-Bezerra demonstrating the preparation of sticky traps; **d** Gad Baneth preparing a bone marrrow smear; **e** Jairo Alfonso Mendoza-Roldan showing a lizard with ectoparasites; **f** Alicia Rojas showing some slide-mounted helminths; **g** Domenico Otranto introducing the course; **h** Filipe Dantas-Torres looking for ticks; **i** Domenico Otranto examining a dog for *Thelazia callipaeda*; **j** Emanuele Brianti and Antonio Varcasia during practical activities on zoonotic canine tapeworms

Last year, in the ninth session of ParSCo, Prof. Petr Volf (Fig. 2a) and Prof. Guadalupe Miró (Fig. 2b) contributed with two different subjects that are highly pertinent to the Mediterranean Basin, *Leishmania* spp. and sand flies and the clinical manifestations of vector-borne pathogen infections, respectively. The first topic addressed the life cycle, taxonomy and clinical manifestations of *Leishmania* spp. from the Old and New Worlds. Then, sand fly ecology, classification and collection methods were discussed followed by the preparation of sticky traps by Dr. Marcos Antonio Santos-Bezerra (Fig. 2c). Sticky and light traps were placed in areas with known occurrence of sand flies, based on a previous study [9]. On the next day, these traps were collected and thoroughly examined for the presence of sand flies and other dipterans. In another course, Prof. Miró explained the most important manifestations of ehrlichiosis, anaplasmosis, dirofilariasis, hepatozoonosis, babesiosis, leishmaniosis and bartonellosis. She discussed five clinical cases she treated at the Clinical Veterinary Hospital of the Universidad Complutense de Madrid, Spain, with the students. In addition, she examined and sampled dogs with suspicion of leishmaniosis, thelaziosis and scabies, explaining the most important clinical signs of these infections and giving practical advice for taking samples from different anatomical locations. These clinical sessions were held in the previous ParSCo courses by Prof. Gad Baneth (Fig. 2d) (Koret School of Veterinary Medicine Hebrew University, Rehovot, Israel) who also involved attendees in the examination of patients and analysis of lymph node aspirates, skin preparations and blood samples.

As mentioned above, parasitology requires constant updating since taxonomic classification of organisms may change with new molecular, ecological and morphological findings [25]. In addition, diagnostics such as DNA-based techniques may be developed with improved sensitivity and specificity as well as simplified accessibility [26, 27]. However, interestingly, the popularity of pets may change, like for reptiles and amphibians; thus, updates on their associated parasites and potential role as reservoirs should be studied. For this reason, Dr. Jairo Alfonso Mendoza-Roldan (Fig. 2e) introduced the most important parasites that can affect these animals and the zoonotic risk they hold. He captured lizards and non-venomous snakes from the field and showed how to draw blood samples and identify key inner organs and the arthropods associated with them. Furthermore, Prof. Alicia Rojas (Fig. 2f) introduced an important area of parasitology, equally diverse and complex, which is helminthology, focusing on worm species that are of zoonotic concern [28]. Moreover, she discussed the main species of nematodes, trematodes and cestodes with an illustrative and practical approach. Participants also had the opportunity to observe specimens and identify eggs, larvae and adults of main helminth species. Although helminths are perceived as far from the concept of vector-borne diseases, the founders of ParSCo, Prof. Domenico Otranto (Fig. 2g) and Prof. Filipe Dantas-Torres (Fig. 2h), discussed the importance of vector-borne nematodes (e.g. *Thelazia callipaeda* and *Dirofilaria* spp.) during the course. Participants had the opportunity to go to the field to look for *Thelazia callipaeda* and its vector, the lacryphagous fly *Phortica variegata* [15] (Fig. 2i). Other helminths addressed during past sessions of the course include feline lugworms and canine zoonotic tapeworms (Fig. 2j).

**Fig. 3:**
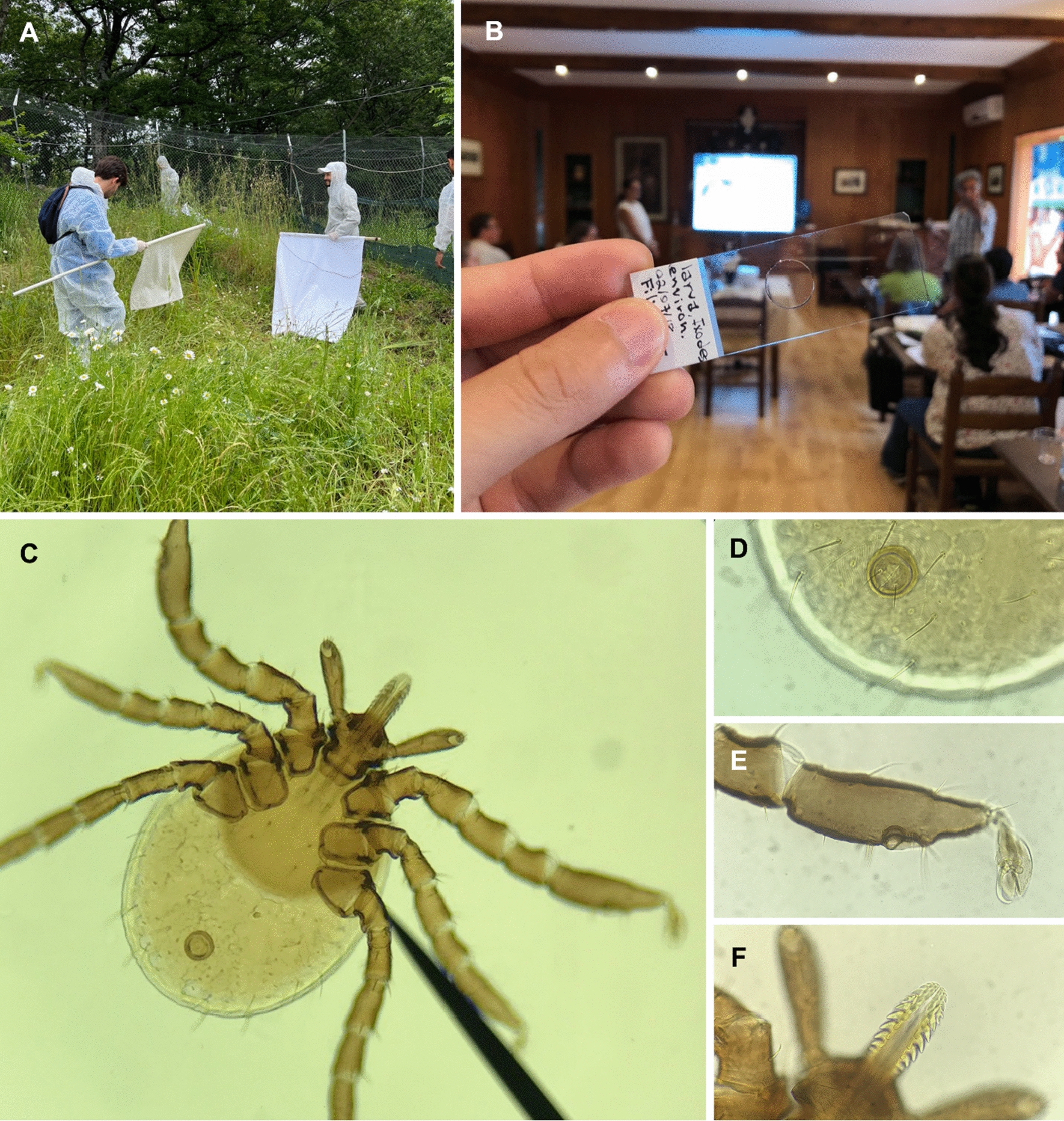
Participants collecting ticks by flagging (**A**). A Slide-mounted larva (**B**) collected by one of the course participants and mounted in Hoyer’s medium by Filipe Dantas-Torres. The general view of the larva of *Ixodes ricinus* (**C**) and some of the morphological strutures presented during the practial lecture on tick anatomy (**D** anus; **E** first tarsus with the Haller’s organ; **F** denticles of hypostome)

Finally, ticks constitute one of the main subjects discussed during ParSCo. Participants can collect, mount and identify the main tick species present in the region, including the castor bean tick, *Ixodes ricinus* (Fig. 3) [6].

Overall, all the sessions of ParSCo have followed the same philosophy, allowing participants to have practical experience in the field of parasitology, being able to see, touch and collect the main parasites present in the Mediterranean Basin. The nine sessions of ParSCo have succeeded in prioritizing practical activities while also having lecturers that showcased their area of expertise in very illustrative and engaging ways.

## A journey into the Basilicata culture and gastronomy

Besides the scientific part, attendees can embark on a unique journey into the Basilicata culture and gastronomy. Basilicata, originally named Lucania, is a mountainous region in the southern part of Italy that straddles the Tyrrhenian and Ionian coasts and has a long history dating back to ancient times. The region has been inhabited by various civilizations, including the Greeks, Romans and Byzantines. This historical significance is evident in the archeological sites, ruins and cities found throughout the region, like the *Sassi di Matera*, a UNESCO World Heritage site since 1993. These structures consist of cave dwellings located in the rocky cliffs, which are one of the oldest continuously inhabited human settlements in the world. Besides the *Sassi di Matera*, Basilicata has several beautiful towns and architectural gems like Maratea, a picturesque coastal town, known for its historic center and the imposing statue of Christ the Redeemer overlooking the sea. Another example is Venosa, the birthplace of the Roman poet Horace, which boasts ancient ruins, including a well-preserved Roman amphitheater. The Norman-Swabian Castle in Melfi and Cathedral of San Gerardo in Potenza are other notable architectural sites. In addition, three national parks surround the ParSCo location in Basilicata and Calabria (i.e. Sila, Pollino and Cilento National Parks).

Basilicata celebrates a range of traditional festivals that have religious origins and feature processions, music, traditional costumes and culinary specialties. Examples include the liturgical feasts of San Gerardo Maiella in Matera and Santa Maria Maggiore in Muro Lucano and the procession of the Misteri in Potenza.

**Fig. 4:**
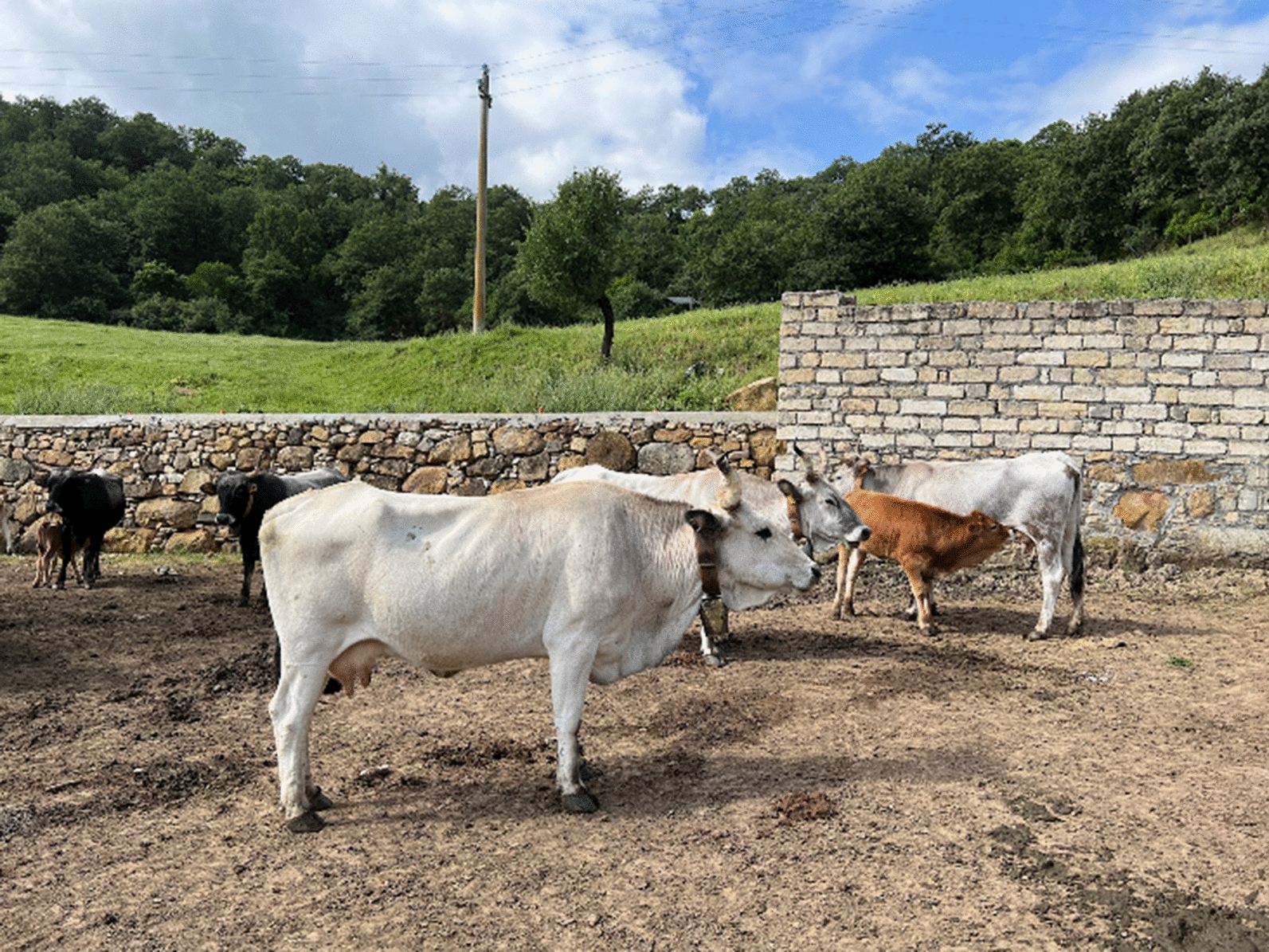
Some Podolica cattle in a typical rural property in the Parco Regionale di Gallipoli Cognato e delle Piccole Dolomiti Lucane, visited during ParSCo. These cattle are allowed to roam freely in the surroundings and are hosts for many species of endo- and ectoparasites

Basilicata’s gastronomy is an integral part of its cultural identity and has traditional dishes rooted in the use of seasonal and local ingredients and techniques passed down through generations. The region offers a range of dishes that highlight its agricultural heritage, including homemade pasta like *orecchiette*, *lagane e ceci* (flat pasta with chickpeas), *fusilli* and *strascinati*. Meat dishes are prominent, with specialties such as lamb, pork, wild boar and especially the meat products obtained from the autochthonous bovine breed of the region named Podolica (Fig. 4). Basilicata is also renowned for its excellent cheeses, bread and desserts like *mustazzoli* and *calzoncelli*. Not to be forgetten, it is also known for its wines, including the *Aglianico del Vulture*.

In essence, Basilicata is not the most known region of Italy, but it is a land that maintains the traditions inherited for centuries in its culture, architecture and gastronomy, with landmarks that are not found in other areas, adding value to the uniqueness of the region.

## Attendees’ presentations: building our community

At the end of every ParSCo, the attendees are invited to showcase their research at the “It’s your turn: attendees’ talks” session, in which they deliver a short presentation about their main research activities and interests. This gives attendees an opportunity to present their research and interests, paving the way for future collaborations among themselves and with the ParSCo team. In fact, these collaborations have helped us to build a large community of parasitologists, which has generated many research projects and publications, contributing to the advancement of veterinary parasitology.

Attendees can also meet key opinion leaders or eminent scientists who have greatly contributed to the field of parasitology, including Prof. Chris Arme, the founding editor of *Parasites and Vectors*. The discussion is often about their future and the perspectives they may have in the field of parasitology. This year, in the 10th session, the attendees will meet Prof. Frederic Beugnet (Boehringer-Ingelheim) and Dr. Bettina Schunack (Elanco Animal Health GmbH), who will share their experience with them regarding how to take the first steps toward pursuing an industry career.

## Celebrating 10 years of ParSCo!

**Fig. 5:**
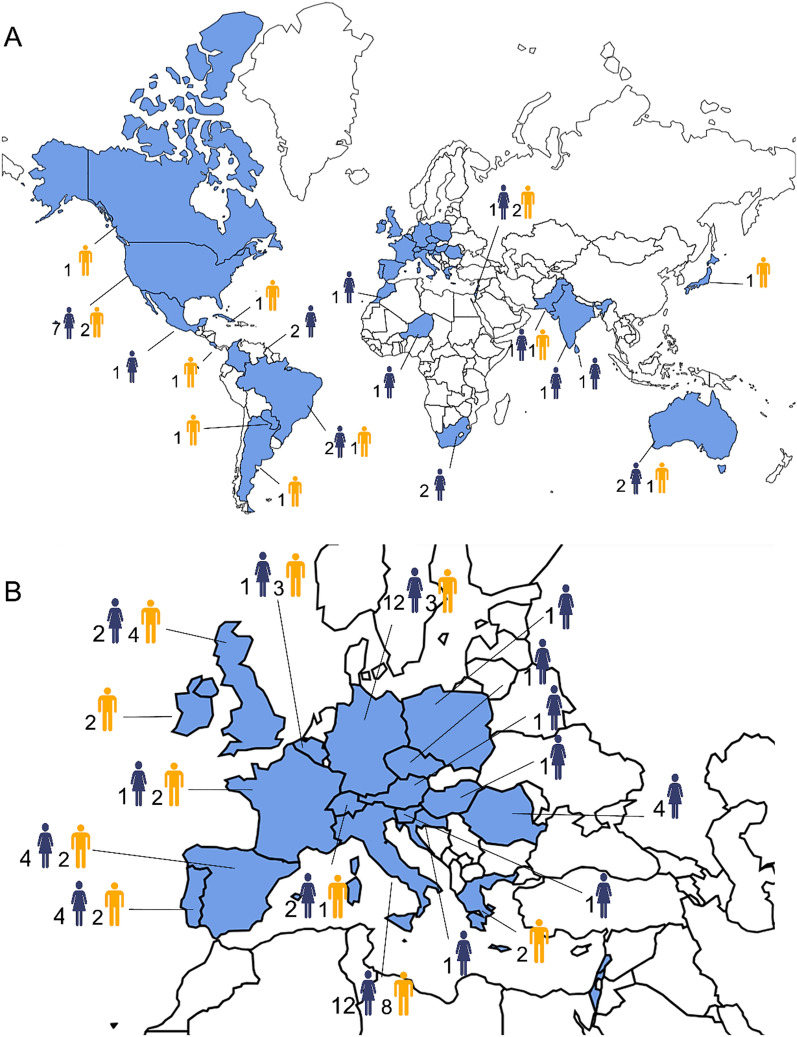
Countries of provenance (**A**, world; **B**, Europe) of attendees from the 1st to 10th sessions of ParSCo

Since the first course back in 2012, over 120 attendees from all continents have attended ParSCo (Fig. 5). At the beginning of this course, the initiators (D.O. and F.D.T.) intended to share their research experience with junior researchers interested in parasitology. It was also an opportunity to continue working together after 4 years of fruitful and intense collaboration. However, over the years, probably because they got older, D.O. and F.D.T. realized that ParSCo allowed them to create a large community of researchers from around the world. For many attendees, it was an opportunity to commence their career, as in the case of Dr. Jairo Alfonso Mendoza-Roldan, a Colombian researcher who is now a permanent member of the academic staff of the University of Bari. Many attendees have become veterinary parasitology specialists and professors of parasitology in different international institutions.

As we celebrate 10 years of ParSCo, we look forward to receiving applications for the forthcoming sessions of this unique summer course, which also provides a great opportunity to become part of a large global network of research collaboration in the field of parasitology.

## Data Availability

Not applicable.
